# Health adaptation policy for climate vulnerable groups: a ‘critical computational linguistics’ analysis

**DOI:** 10.1186/1471-2458-14-1235

**Published:** 2014-11-28

**Authors:** Bastian M Seidel, Erica Bell

**Affiliations:** Graduate School of Medicine, University of Wollongong, Northfields Avenue, Wollongong, New South Wales 2522 Australia; Huon Valley Health Centre, 85 Main Road, Huonville, Tasmania 7109 Australia; University Department of Rural Health, University of Tasmania, Private Bag 103, Hobart, Tasmania 7001 Australia

**Keywords:** Health adaptation policy, Health equity, Unequal health outcomes, Climate vulnerability, Social determinants of health

## Abstract

**Background:**

Many countries are developing or reviewing national adaptation policy for climate change but the extent to which these meet the health needs of vulnerable groups has not been assessed. This study examines the adequacy of such policies for nine known climate-vulnerable groups: people with mental health conditions, Aboriginal people, culturally and linguistically diverse groups, aged people, people with disabilities, rural communities, children, women, and socioeconomically disadvantaged people.

**Methods:**

The study analyses an exhaustive sample of national adaptation policy documents from Annex 1 (‘developed’) countries of the United Nations Framework Convention on Climate Change: 20 documents from 12 countries. A ‘critical computational linguistics’ method was used involving novel software-driven quantitative mapping and traditional critical discourse analysis.

**Results:**

The study finds that references to vulnerable groups are relatively little present or non-existent, as well as poorly connected to language about practical strategies and socio-economic contexts, both also little present.

**Conclusions:**

The conclusions offer strategies for developing policy that is better informed by a ‘social determinants of health’ definition of climate vulnerability, consistent with best practice in the literature and global policy prescriptions.

## Background

### Rationale and aim

The fourth assessment report of the Intergovernmental Panel on Climate Change defined adaptation as an ‘Adjustment in natural or human systems in response to actual or expected climatic stimuli or their effects, which moderates harm or exploits beneficial opportunities’ —in contrast to mitigation which refers to action to reduce carbon emissions [1]. An important opportunity now exists to build the foundations for better health adaptation to climate change for climate vulnerable groups, as many countries move to develop national adaptation policy statements, including for health. Twelve Annex 1 (‘developed’) countries in the United Nations Framework Convention on Climate Change [2] have produced national adaptation policy and planning documents in English. Only five of them have specific health adaptation policies. Our previous research used a qualitative approach to identify the dominant discourses of knowledge in such policies: scientific and epidemiological empiricism and public sector operationalization. It explored how such policy documents privilege particular kinds of knowledge, research methods and evidence about local community knowledge and applied and health services research.

The aim of this paper is to consider answers to a different question: how are specific climate vulnerable groups represented in adaptation policy? What are the implications of this for best practice in developing policy for such climate vulnerable groups? The examination of adaptation policy documents and the modelling of best practice for such policy development, particularly for climate vulnerable groups, is a neglected area. What can be argued is that the climate and health field faces considerable challenges of evidence translation: despite the large corpus of climate and health research, there is still relatively little applied *health* adaptation happening at the coalface of local community level [3–9]. Despite a body of climate and health research now numbering over 6,000 articles and reviews in health science journals, most published since 2005, public health policy-makers and leaders feel unprepared and unable to ensure health systems make appropriate adaptations [10].

This paper builds on previous work in the field to offer two kinds of contributions. The results section maps the extent and nature of existing national adaptation policy for climate vulnerable groups in an exhaustive sample of 20 policy documents from 12 countries. The conclusion section identifies the implications of these findings in the light of emerging best practice in adaptation informed by a ‘social determinants of health’ definition of climate vulnerability. Practical strategies are offered for best practice in developing the purpose, processes, content and structure of national adaptation policy, including participative processes for climate vulnerable groups. In so doing, this study offers not simply findings about what is lacking in adaptation policy but also a concluding practical discussion of how to develop better health adaptation policy for these climate vulnerable groups.

An acknowledged limitation is the focus here on developed countries. An examination of the policy complexities in developing countries is beyond the scope of this study and must have separate consideration. It is hoped that in focussing on the developed or ‘Annex 1’ countries that, under the United Nations Framework for Climate Change, were intended to lead the way in producing adaptation policies, this paper will make a contribution to knowledge about how well these countries are delivering on that duty [2].

### Defining climate-health vulnerability and deciding vulnerable groups

As defined by the 2014 5th Assessment report of the Intergovernmental Panel on Climate Change, vulnerability is seen in this study as ‘The propensity or predisposition to be adversely affected. Vulnerability encompasses a variety of concepts including sensitivity or susceptibility to harm and lack of capacity to cope and adapt.’ Contextual vulnerability or ‘starting-point vulnerability’ refers to ‘A present inability to cope with external pressures or changes, such as changing climate conditions. Contextual vulnerability is a characteristic of social and ecological systems generated by multiple factors and processes’ [11]. This study does not assume that all effects of climate change are negative: in some regions already disadvantaged groups may benefit from climate change, while climate change may affect new groups.

This study further defines climate-health vulnerability as a form of health vulnerability arising from interactions between climate and socioeconomic disadvantage to exacerbate already unequal health outcomes for particular groups that may be additionally physically, intellectually, culturally or geographically disadvantaged. In this study, this definition has been used to identify nine groups that are not mutually exclusive: people with mental health conditions, Aboriginal people, culturally and linguistically diverse (CALD) groups, aged people, people with disabilities, rural communities, children, women, and those who are socioeconomically disadvantaged. The justification for this definition and for including these nine groups in the present study is as follows.

A large body of evidence has accumulated to suggest that health is strongly influenced by socioeconomic factors: that health is distributed in ways that are closely linked to the particular social and economic conditions of people’s lives [12, 13]. This does not mean that the social determinants of health are the only primary health determinants. However, the ‘social determinants of health’ have been described as the risk factors that work at the social, not individual, level to determine the risk for a disease, such as Type II diabetes. The social determinants of health can therefore be understood as the primary determinants of health: ‘In countries at all levels of income, health and illness follow a social gradient: the lower the socioeconomic position, the worse the health’ [14]. These socioeconomic determinants are in fact a constellation of factors that are not restricted to such obvious economic shapers of health as ‘income’ and ‘unemployment’. They include factors such as disability, poor health literacy and cultural differences in attitudes to health that shape the ways people access services and their lower health service utilisation, for example, in rural and Aboriginal communities [14, 15].

Climate vulnerability also has a known strongly socioeconomic basis. It has been described as exacerbating the existing socioeconomic root cause of unequal health outcomes to further increase health inequalities [16]. While the body of literature on this suggests that socioeconomic factors act in complex ways with other individual, local and global factors, there is sufficient evidence to support the view that an engagement with those climate vulnerable groups that are also experiencing social disadvantage should be an adaptation policy priority. The climate change literature most frequently refers to the following nine groups: people with mental health conditions, Aboriginal people, CALD groups, aged people, people with disabilities, rural communities, children, women, and people who are socioeconomically disadvantaged [17, 18]. These vulnerable groups often share one or more of the risk factors described in the applied literature on risk assessment for climate-vulnerable groups: unequal access to education and lower literacy; unequal health outcomes; susceptibility to chronic and/or infectious diseases; poorer nutrition; lower life expectancy; lesser access to health services; lower health literacy; gender inequality; unemployment; lower income; poorer quality housing; concentration in ‘at risk’ geographic areas with poorer community and public health infrastructure supports; lesser water quality and supply; exclusion from decision-making processes; lesser social and civic participation [19]. As the 2014 Fifth Assessment Report (AR5) of the IPCC suggests, those who are socioeconomically disadvantaged in wealthy, not only poor, countries will bear the heaviest burden of climate change. Climate change works to increase existing inequalities and make it more difficult for those who are already socially, economically or culturally disadvantaged or excluded to overcome that disadvantage [11]. Accordingly, the rationale for this study is given by the broader definition of vulnerability to disasters and emergencies provided by the World Health Organisation, which suggests why health adaptation policy should prioritise those most ill-equipped to adapt:

Vulnerability is the degree to which a population, individual or organization is unable to anticipate, cope with, resist and recover from the impacts of disasters […] Vulnerability is a function of susceptibility (the factors that allow a hazard to cause a disaster) and resilience (the ability to withstand the damage caused by emergencies and disasters and then to recover) […] The concept of vulnerability helps to identify those members of a population who are most likely to suffer directly and indirectly from a hazard. It is also useful in identifying those who are more likely to suffer longer-term disruptions of livelihoods and life-lines, as well as those who will find it more difficult to re-establish their accustomed patterns of living […] This has important implications in defining priorities for vulnerability reduction [20].

However, while the nature of climate vulnerability is broadly known, as are specific climate vulnerable groups, no scholarly paper has to date been published on the extent to which the emerging body of national adaptation policies engages with the climate-health vulnerabilities of specific groups—the focus of this paper. Before such an examination can be made it is necessary to consider global policy prescriptions for best practice in adaptation.

### Global policy prescriptions for climate vulnerable groups

The section examines the answer to this question: What are the global and intra-country prescriptions for adaptation policy, *particularly for health*, which frame national policy efforts? The reply to this question, which has not been provided elsewhere in the literature, is necessarily descriptive to provide the evidence for an argument critical to understanding the nature and importance of this study’s findings. The discussion here is not a history of the development of policy documents—a far more complex task than can be attempted in this section—but rather an attempt to sketch the answer to this specific question. In a sentence, this section demonstrates that global policy prescriptions for best practice heavily emphasise engagement with the needs, experience and knowledge of vulnerable and disadvantaged groups.

The United Nations Framework Convention on Climate Change (‘the UN Convention’), ratified by 195 (now 196) Parties to the Convention, came into force in 1994 and placed a strong onus on developed countries to lead the way in climate change action [2]. While Article 4 required countries to adopt mitigation and adaptation plans (nationally and regionally through intracountry agreements), adaptation received a lesser emphasis than mitigation initially [2] until the release of the Third Assessment Report of the Inter-Governmental Panel on Climate Change provided greater impetus and an evidence base for adaptation responses [21]. However, the Third Assessment Report of the IPCC should not be considered the primary impetus for including adaptation as one of the pillars of the negotiations under the UNFCCC, although it is likely that the assessment was helpful to the discussions.

At the time of writing, the key international policy document for the UN Convention is the Cancun Adaptation Framework as part of the Cancun Agreements [22] that provide the policy framework for the work of the Adaptation Committee, which aims to provide an integrated approach to adaptation. The Cancun agreement asserts that adaptation must be given the same priority as mitigation (I.2 (b)). It emphasises engaging with and learning from the knowledge of climate vulnerable communities, including Aboriginal communities, at the local level, for effective climate change responses (I.7) (II.12). It also asserts the need for a process for least developed countries to produce national adaptation plans [22].

The Cancun Adaptation Framework was substantially advanced at the 2011 Durban Climate Change Conference which produced a further policy framework for the development of National Adaptation Plans for developing countries [23] based on National Adaptation Programmes of Action. The National Adaptation Programmes of Action, while not classified here as policy documents, should be noted as providing a process for ‘Least Developed Countries’ to identify priority activities that are urgent adaptation responses to climate change; 47 countries have completed these to date [23]. The Durban framework [23] also reinforces the role of climate vulnerable, including Aboriginal, communities, and their knowledge, and the importance of intersectoral integration and local-level participation and approaches in the UN Convention [2].

Concurrently—and in line with Article 4.1 (b) and (e), of the UN Convention—adaptation guidelines and reporting to the UN under the Convention have placed relatively little emphasis on health. All Parties to the Convention are required to produce regular national communications that include a section on adaptation. However, while 42 (Annex 1 or developed) countries/country entities have submitted reports due on 1 January 2010 (‘Fifth National Communications’) and 44 have submitted reports due on 1 January 2014 (‘Sixth national communications’), these provide little by way of statements of policy or information about specific groups affected by climate and health vulnerabilities [24]. Notwithstanding, it is likely that national health agencies will increasingly call for a greater focus on health in the national communications—adaptation generally will continue to receive greater attention in the national communications of these developed countries. A further 102 (non Annex1) or developing countries have submitted at least two national communications, and 6 have submitted three national communications under the UN Convention. Of course, although little specific climate and health information about climate vulnerable groups is included, reporting on other sectors such as agriculture takes a similar broad or overview perspective.

The World Health Organisation’s work on climate change and health is consistent with the fundamental prescriptions of the UN Convention for adaptation best practice. The WHO’s policy position on climate change adaptation is articulated through World Health Assembly and Executive Board policy documents on climate change and health, notably WHO resolution WHA61.19 [25] and the WHO workplan outlining the WHO’s policy priorities for climate and health actions based on that resolution [26]. The resolution gives primacy to ‘vulnerable local communities’ and ‘strengthening health systems’ and describes climate science as delivering understandings of ‘potential consequences’ but not necessarily adaptive responses (just as the UN policy does not position climate science as offering a foundation for community engagement). For example, items 1.(4) and 1.(5) place a strong emphasis on applied research and evaluation (‘decision-support and other tools’) in adaptation research for impacts and risks. Items in section 2 focus on local health service capacity-building [25]. The workplan reflects the community-based, applied, health services research, and practical tools focus of the resolution [26], and in so doing both are consistent with the policy of the UN.

The 2008 WHO resolution for action on climate change health risks [25], adopted by the 193 countries of the World Health Assembly, led to regional frameworks of action for each of the six WHO regions. The policy of the WHO has therefore now also been articulated through five WHO regional committee resolutions and associated regional frameworks [27–31] (no such regional policy information is displayed on the WHO website for the Americas). The WHO regional frameworks, such as the Europe framework, articulate a vision of climate vulnerability informed by local ‘health system preparedness’ and community-based, regionally responsive applied or operational research methods, as in Objective 2: ‘Strengthen health, social and environment systems and services to improve their capacity to prevent, prepare for, and cope with climate change’ [28]. Fundamentally, this is a policy vision of best practice for health adaptation underpinned by local community engagement.

The European Union’s (EU) international approach to supporting developing and vulnerable countries offers further evidence that global frameworks are also less about climate science and more about local community engagement, particularly for vulnerable groups. The EU’s work is also shaped by the UN Convention [2]. However, neither the European Environment Agency nor the European Commission has developed a policy framework or even basic agreed-on definition for national adaptation strategies for Europe. Yet a white paper providing a European framework for action adopted by the European Commission was published in 2009 [32]. The document devotes a one page section to human health and social policies that takes a strong focus on social justice and vulnerable populations: adaptation policies must ‘distribute the burdens equitably’. Notwithstanding, this concern for vulnerable groups is located within the wider economic and natural resource management focus of the document in which economics is made a key case for ‘a strategic approach to adaptation’ [32]. It is also worth noting that a 2010 European Environment Agency report also refers to an intention to publish a Communication on Mainstreaming Adaptation and Mitigation in 2011 and a comprehensive EU adaptation strategy to be developed by 2013 [3].

How well have such broad global prescriptions for addressing the needs of climate vulnerable groups, in ways that include these communities’ experiences and views and address their practical health service needs, been operationalized in national adaptation policy? To answer this question, we must first identify what are national adaptation policy documents and a method for analysis of their content.

## Method

### Research questions

The specific research questions were: ‘What kinds of content define adaptation in national policy documents that have government jurisdiction over the health sector?’ and ‘How is content about nine known climate vulnerable groups related to this content?’

### Study sample

While there are a number of surveys of adaptation policy for particular regions such as Europe, [4, 5] quantification of the presence of references to specific vulnerable groups has not been offered in a global survey of all current national adaptation policies for *health*. Accordingly, the study sample represents a universe of national adaptation policy documents that have jurisdiction over the health sector, whether they are health specific or not. It includes 20 policy statements from 12 countries, including five countries with six health-specific national adaptation statements, as of March 2012 (with the exception that, after this date and before acceptance of this paper, one document for the Netherlands available in March 2012 [33] was no longer publicly available and was replaced by another [34], so this alternative policy statement was substituted accordingly, in line with the sample criterion that documents be publicly available). The following provides the sample numbers of general versus health-specific policy documents, by country, including relevant citations with available URLs: *Australia* has two documents, one general [35] and one health–specific document [36]; *Belgium* has one [37]; Denmark has one [38]; *Finland* has one [39]; *Germany* has one [40]; *Netherlands* has two [35, 41]; *Russian Federation* has one [42]; *Scotland* has one general [43] and one health-specific document [44]; *Spain* has one [45]; *USA* has one health-specific document [46]; *United Kingdom* has four, including two general documents [47, 48] and two health-specific documents [49, 50], the latter being an update of the former; *Wales* has two general documents [51, 52] and one health-specific document [53].

Our method in obtaining this exhaustive sample of national health adaptation policy statements has been described in our previous study and will be summarised here. The sample included national adaptation policy guidelines and planning documents by government agencies, in English, available from all 42 Annex 1 (‘developed’) countries of the United Nations Framework Convention on Climate Change [2]. The websites of all health and environmental agencies in all these 42 countries were searched, with follow-up requests for publicly available material made to those agencies.

The definition of a ‘national adaptation policy document relevant to health’ used in this study is suggested by the inclusion criteria used for the sample search (exclusion criteria are given in parenthesis):

- ● publicly available statements in English (not any other language) from sovereign and non sovereign countries
- ● statements from government agencies (not non-government agencies such as professional associations)
- ● policy and planning documents (not implementation documents)
- ● climate adaptation documents (not mitigation documents, unless both adaptation and mitigation policies were intended to be covered in a single national document)
- ● general climate adaptation policy documents (not policy documents dedicated to single issues such as heat health plans)
- ● substantial policy documents (not, for example, brief statements such as letters of personal commitment from senior bureaucrats sometimes published on agency websites)

In relation to sample limitations, a determination of the extent of health adaptation policy documents not in English is beyond the scope of this study and cannot be accurately made without very substantial language translation resources for searching for, translating and analysing such documents. Further, the exclusion of implementation documents meant that the study did not focus on the detail of how such policy was interpreted and reinterpreted by communities or what communities have done in the absence of such policy. The extent and quality of local initiatives suggested by policy documents in English for Annex 1 ‘developed’ countries also cannot be assumed to be greater than what may be in place in non Annex 1 countries at the local level. Such an assumption may be wrong not simply because policy documents are not a measure of local creativity anywhere, but also because there is a substantial body of emerging literature suggesting that local indigenous cultures bring sophisticated and creative adaptive capacities and strategies to a climate-changing world [54–61].

### Analytic procedure

The study method is informed by the belief that combining elements of two ostensibly opposed methods—‘computational linguistics’ and ‘critical discourse analysis’—can offer broad, reproducible and accurate findings about the quantifiable content of a large language dataset as well as insights about what it values, how, to extend the empirical findings. In order to balance requirements for objectivity with the need for nuanced readings of policy texts, computational linguistics software [62] is used here to offer objective, machine based findings about the presence of explicit references to vulnerable groups. These findings are the basis for the conclusions. However, machine-based findings are supplemented with critical discourse analysis offering nuanced readings of the text that necessarily involve interpretation, implemented within the established paradigm of textual exegesis that is critical discourse analysis [63–66]. The analysis of the 20 national adaptation policy documents can therefore be described as using a novel ‘critical computational linguistics’ method with two stages:

#### Stage 1 Quantitative content analysis

The key findings of the study rest on the machine-driven, reproducible procedures from this stage. This stage involved software-driven quantitative mapping of the frequency and co-occurrence of all concepts found using automated procedures in the national policy documents (48 such automated concepts were found). The analysis also included an additional nine researcher-selected concepts for climate vulnerable groups: people with mental health conditions, Aboriginal people, CALD groups, aged people, people with disabilities, rural communities, children, and women, and those who are socioeconomically disadvantaged. This stage aimed to identify the degree to which this total of 57 concepts were present and relationships between them—effectively a map of relationships between all key concepts in the documents found by automated methods and any content about the nine researcher selected climate-vulnerable concepts. Software developed for this purpose known as Leximancer [62] was used for this first content analysis stage. Detailed discussion of its technical features and basis in Bayesian approaches to computational linguistics is provided in a validity study [67]. Fundamentally, the software operates in an iterative manner to visualise language data as a set of connections between either machine-selected or/and researcher-selected concepts. The software produces a concept map that visually represents the relative frequency of concepts in the language dataset and their overall contextual proximity i.e. their nearness to one another when all concepts are considered. A simple clustering algorithm was used that was rerun 10 times (the concept map is stochastic). The software has been applied across multiple disciplines cited in our previous work applying the method of application of Leximancer described in this paper.

**Figure 1:**
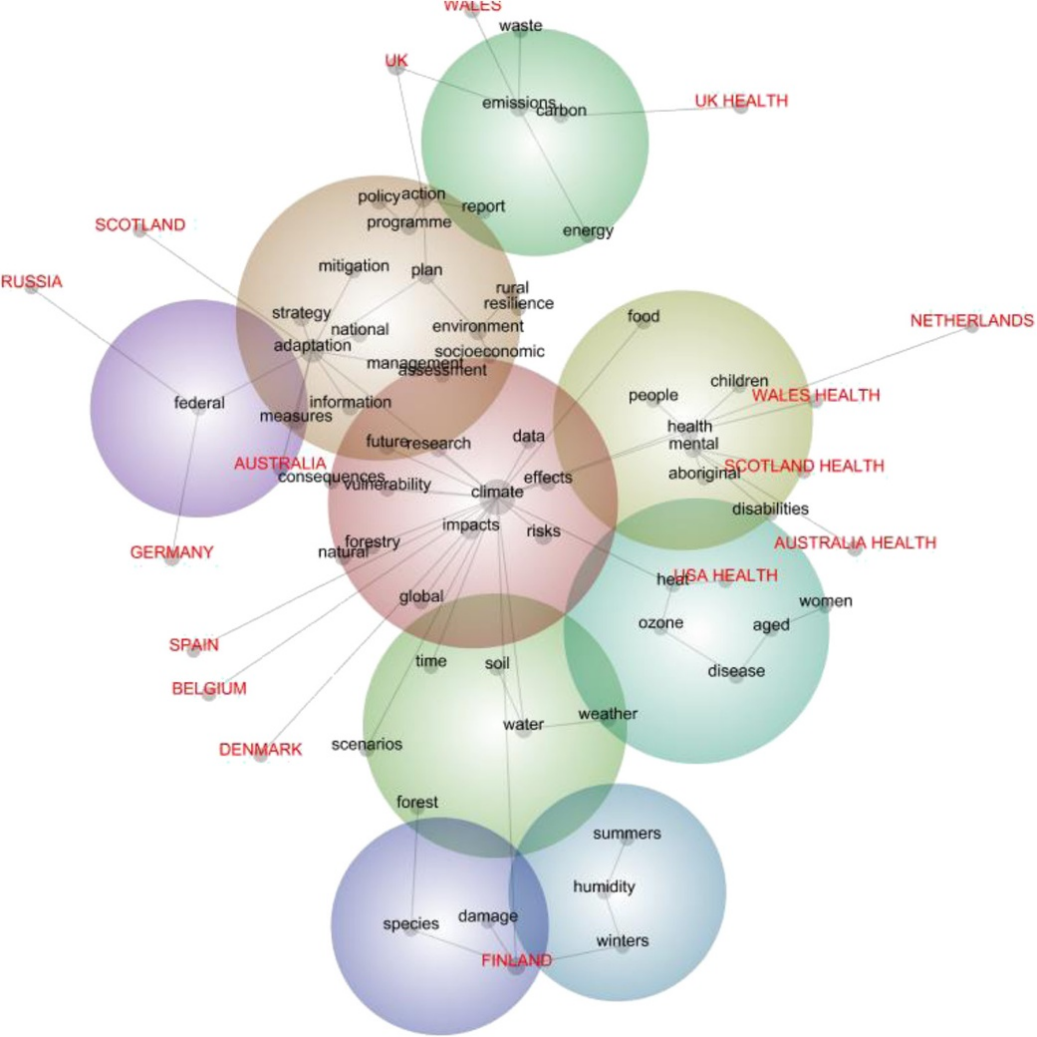
**Concept map of adaptation policy documents from 12 countries: all 57 concepts comprising the content of documents and their relationship to eight vulnerable groups concepts found; proximity to all concepts of individual country documents is also shown (split by whether health or general adaptation documents).**

In this study, the set of 57 concept words (48 found and nine researcher selected) was used to explore relationships between all key content in the document and other content about the climate vulnerable groups. The 48 concepts found to represent the entire conceptual structure of the documents (i.e. the whole dataset) by the automated concept mapping procedure in Leximancer are shown in Figure 1. The rationale for the additional nine researcher selected concepts for climate vulnerable groups is given in the definitions section. These nine concepts are also shown in Figure 1 with the exception of the CALD concept which was not found at all in these documents.

The unit of analysis in Leximancer is text blocks that are about a paragraph in length. A text block may contain one or more concepts. In this language dataset, there are 38,903 instances of concepts in 9,769 distinct text blocks. At about 3 text blocks per page that equates to 3,256 pages of policy text in the total dataset or the equivalent of about 13 books comprised of 250 pages each. Given the analysis is of all content of the policy documents, this is the size of all the policy documents collectively considered.

It should be emphasised that Leximancer includes multiple text windows and raw data lists for instantaneous access of the text blocks that form the basis for a concept. For example, while viewing the concept map, the researcher can access not only all text blocks that form the basis of individual concepts, but also the context or original policy document from which each text block within a concept was extracted. This allowed an iterative process of constant checking of the analysis to ensure that the method had not produced any significant omissions or inconsistencies. It also meant that the qualitative discourse analysis in stage 2 could be built on the dataset selected in stage 1 and undertaken in ways that involved iterative checking of all references to vulnerable groups, in context.

#### Stage 2 Qualitative analysis of text blocks about specific vulnerable groups to examine dominant discourses

This stage involved qualitative analysis of all text blocks selected by the software for the climate vulnerable groups with sufficient content about them to be included in the analysis i.e. again, excluding the CALD concept because it was not found in any text blocks. Therefore, eight groups were the focus of this analysis: women, people with disabilities, people who are elderly, Aboriginal people, children, people with mental health issues, rural communities and people with socioeconomic disadvantage. This stage was informed by a theoretical approach now widely used in qualitative health research—critical discourse analysis—which allowed exploration of the domination discourses or language practices in the policy references to climate vulnerable groups. In such an approach, the focus is upon language as a technique of power that variously represents or marginalises the interests of different social groups [63–66]. This analysis focussed on the ways that the language of policy works to ‘normalise’ (make seem natural) certain assumptions about climate vulnerable groups in the dominant discourses or language practices for representing these groups. Thus, the critical discourse analysis in this study adds possible theoretically informed explanations of how to understand the nature of policy in ways that go beyond frequencies and counts of references. For example, we could hypothesise that even if references to some vulnerable groups are far more numerous than references to other vulnerable groups, both sets of references may use similar language practices to reproduce similar ways of marginalising the interests of both groups in a dominant discourse within and across references to these groups. Accordingly, critical discourse analysis should be viewed as an extension hypothesis-building analysis that aims to enrich the machine-driven quantitative analysis by identifying dominant language practices.

In summary, in this study, computational linguistics has been used to offer reproducible and accurate quantification of language upon which broad, scientifically acceptable findings can rest. The key findings of the study are established by the Leximancer concept map and its supporting quantitative data to offer broad, reproducible conclusions about 1) what kinds of content define adaptation in national policy documents and 2) whether there is content about nine known climate vulnerable groups and how this content is related to all other content in the policy documents.

## Results and discussion

### The concept map

This section provides detailed discussion of the supporting data for the concept map. However, not all readers are quantitatively minded. For these readers, Figure 1 offers a visual picture or snapshot of these semantic relationships: where concepts are located one in relation to another, where the most frequent concepts tend to be and the typical semantic pathways between them. Accordingly, Figure 1 provides a map of the concepts in the adaptation policy documents, including the selected concepts for climate vulnerable groups. The words on the map are written in different typefaces (capitals versus lower case) and colours (red type versus black type) to distinguish where country documents (red capitals) are placed in relation to all conceptual context (black lowercase). The documents grouped by country (in red capitals), are distinguished by whether a health document or general adaptation document i.e. if a health document, the word ‘health’ appears after the name of the country, therefore, four countries are mentioned twice because those four countries have health-specific documents that are mapped separately, while one country (USA) has only one (health specific) adaptation policy statement. The spatial location of the concepts (and the country document labels) indicates their proximity, taking into account associations between all concepts. The ‘heat-mapped’ nature of the spheres means that degrees of warmth in their colour correspond to the frequency of the concepts inside the sphere. That is, if the most common concepts are in the darkest red spheres (warmest) and the least common concepts are in the purple spheres (coldest) then colours in-between that spectrum (e.g. pale green) reflect concepts that tend to be in-between in terms of their overall frequency. The size of the dots associated with a particular concept indicates frequency of overall co-occurrences between that concept and all other concepts mapped. The grey lines between the concepts and the country names indicate typical pathways across *multiple concepts* i.e. typical storylines.

For example, the spatial location of the eight vulnerable groups concepts is telling (again, no references were found to the ninth concept CALD). It suggests that some concepts about vulnerable groups (children, mental health, Aboriginal people, disabilities, women, people who are aged) tend to be more associated with four kinds of health documents from Wales [53], Scotland [44], Australia [36] and the USA [46] (though no grey line connects the USA document to the Aboriginal concept). Some kinds of vulnerable group concepts are more common than others, for example, references to mental health issues are more common than discussion of people who are aged. In relation to typical storylines, specific references to strategies do not appear well-connected to the climate vulnerable groups or with any other social group. The story that the concept map tells is that, while rural communities are referred to in terms of their resilience, other groups are far more often referred to in terms of climate ‘effects’. The ‘socioeconomic’ concept tends to be more about rural communities than other climate vulnerable groups such as people with ‘disabilities’. The typical storyline for the little present ‘aged’ concept is about ‘disease’. Discussion of ‘plans’ and ‘adaptation’ and ’programmes’ is not well linked to discussion of specific climate vulnerable groups.

These findings have important implications for the extent to which national policy is understood to be consistent with global guidelines for managing climate vulnerability. However, the nature of the concept map needs to be understood when weighing these findings. It is a summary visual picture condensing a great deal of quantitative analysis of the qualitative data. Again, broad observations can be made looking at the map to get a ‘bird’s eye view’ of the quantitative analysis but the details of the quantitative data supporting the map need to be retrieved and examined to further explore the meaning of the map.

### Overall concept frequency

Accordingly, when the quantitative data supporting the concept map are examined further they suggest the following. Seven concepts out of the larger set of now eight climate vulnerable concepts (again, excluding CALD which was not present in these documents) are among the most infrequent concepts in the policy texts: rural, socioeconomic, children, Aboriginal, aged, disabilities, and women which have a 2%, 1%, 1%, 1%, 0%, 0%, and 0% likelihood respectively of being found in any one of the 9,769 distinct text blocks in the dataset. The concept of ‘mental health’ has a 12% likelihood of occurrence relative to all other concepts in these documents. The most common concepts are, not surprisingly, climate, adaptation, impacts, plan, and emissions (100%, 36%, 19%, 16%, and 15% likelihood respectively). The three most common concepts after these are: water (14%), health (14%), risks (13%). These figures confirm what the map in Figure 1 suggests: generally speaking, policy documents are constructing climate and adaptation issues in ways that most often do not refer to climate vulnerable groups, with the exception of people with mental health issues.

### Paired co-occurrences of concepts

Another kind of data produced by Leximancer relates to *paired* co-occurrences not represented on the map in Figure 1 which is about *overall* co-occurrences. For example, the concept word ‘strategy’, which has a 10% overall relative frequency, has the following paired co-occurrences with vulnerable group concepts (likelihood of being found in the same text block): socioeconomic (21%), rural (13%), mental health (9%), people who are aged (5%), Aboriginal people (3%), children (2%). There is not a single instance of the ‘strategy’ concept being paired in the same text block with ‘disabilities’ or ‘women’. It is in fact most likely to be paired with the concept ‘federal’. Again, this presents a story of policy about practical strategies being disconnected from the specific needs of climate vulnerable groups i.e. strategies appear to be represented as homogenous and not distinguished in relation to climate vulnerable groups.

The concept word ‘health’ is, as has been seen, a smaller body of references in this dataset, yet it is in contrast very closely paired with the vulnerable group concepts, as the concept map suggested in Figure 1 in the alignment of health specific adaptation documents. Six of the total set of eight vulnerable concepts found are in the top six paired co-occurrences of concepts for the ‘health’ concept, suggesting an apparent strong equity focus of health-specific adaptation policy: mental health (98%), disabilities (75%), people who are aged (70%), Aboriginal people (62%), children (54%), and women (50%). The ‘health’ concept also co-occurred with the ‘rural’ concept 26% of the time and, interestingly, the ‘socioeconomic’ concept least frequently of all the vulnerability concepts with a 13% paired co-occurrence.

As the foregoing suggests, the socioeconomic concept is poorly related to any of these vulnerable group concepts. Not only that, where it occurs in the 71 text blocks listed for it, its most common paired concept is ‘children’ (3%). The next (8th) most common paired occurrence with ‘socioeconomic’ is ‘rural’ (2% paired co-occurrence). The ‘socioeconomic’ concept is found most frequently n the Scotland health document [44] (2%), the German document [41] (2%), the Wales health [53] (2%) and other general Wales documents [68, 69] (12%). It is not found at all in the USA document [46]. The paired–co-occurrences therefore quantify and confirm what the concept map suggests visually and also offer evidence that the limited presence of the socio-economic concept is generally shared across country documents.

### Critical discourse analysis

A traditional critical discourse analysis allows further analysis of the nature of meanings produced in the references to climate vulnerable groups that lie beyond quantifiable dimensions of language. In the analysis that follows, the key emphasis is upon understanding the story that emerges from the critical quantitative findings about disconnects between references to climate vulnerable groups and references to practical strategies and socio-economic contexts. Space restrictions do not allow exhaustive description of every instance of a reference to a climate vulnerable group—there are 7,970 instances in 1,376 text blocks of references to climate vulnerable groups, out of a total of 38,903 instances of concepts in 9,769 text blocks. Accordingly, Table 1 presents the overall findings of the discourse analysis. It shows, for each climate-vulnerable group, the details of numbers of references in numbers of text blocks (many containing more than one reference), by country, as well as the dominant discourse used in language referring to that group i.e. covering more than three quarters of all references to that group, with citations to illustrative supporting material. That is, Table 1 provides not simply counts: it also explains what are the dominant discourses in the language about each of the vulnerable groups—information that a machine cannot provide. Table 1 also provides references to the specific publicly available policy documents to substantiate the findings of the critical discourse analysis. Overall, the table supports findings from stage 1 and suggests how the policy language works to create the illusion that the needs of vulnerable groups are being considered without articulating practical strategies or socioeconomic causality in ways useful to guiding practical policy implementation. Generally speaking, what the dominant discourses for each of the vulnerable groups have in common is a reliance on ‘lists’ of vulnerable groups that act as a kind of tokenism or ostensible policy inclusiveness. Even the most common vulnerable group concept (mental health) does not move beyond populist and poorly distinguished concepts of mental health climate vulnerability

**Table 1:** **Dominant discourses in the policy language referring to each of eight climate vulnerable groups (excepting CALD which was found to have no references)**

Climate vulnerable group	Number of ***total instances*** of references to that group across all policy documents	Number of ***text blocks with one or more references*** to that vulnerable group, by country, distinguished by whether in health or general adaptation documents	Dominant discourse used in language referring to that group i.e. the definition of the discourse in the box covers three quarters or more of all references to that group across all documents (citations of country examples are given in parenthesis)
Women	4	Belgium (2); Wales (1); Australia (1)(total =4)	Incidental references that do not form a discourse of specific vulnerability except in one reference to women and poverty [53].
People with disabilities	75	Wales health (4); UK health (4) Australia health (1); UK (1); Finland (1)(total =12)	Dominant discourse is of exhortation to include groups in adaptation planning and strategies, in lists of groups, only one of which is people with disabilities [49].
Older citizens	218	Australia health (12); Finland (10); Scotland health (4); Denmark (2); UK health (2); Germany (2); USA health (1); Belgium (1); Wales health (1); Wales (1); UK (1)(total =37)	Dominant discourse is about heatwave vulnerability i.e. vulnerability to heat and ground-level ozone, airbourne allergens, and other pollutants, with the few references to specific adaptation strategies for older citizens being limited to ‘top-down’ solutions, not ‘bottom up’ local knowledge and conditions [39].
Children	378	UK health (21); Australia health (16); Wales health (4); Scotland health (3) Scotland (3); Germany (2); Belgium (2); Finland (6); Australia (1); UK (1); Denmark (1); USA health (1)(total =65)	Children are predominantly referred to in lists of groups affected by heatwaves and other extreme events for which planning is required. However, there are also references to specific environmental health issues for children such as air quality and asthma (DEA, 2008) and mechanisms for achieving climate policy goals involving children, such as education (SG, 2009) for sun smart behaviour (SG, 2010), and reduction of obesity (DH, 2010). With exceptions in Wales and UK documents generally (CCHWG, 2009), most references to children do not include broader economic costs of climate change or allude to generational equity, as in the Scottish general document which is nonetheless silent on the unequal burden on the poorest children (SG, 2009). In contrast to other climate vulnerable groups, there are also allusions in these documents to a lack of knowledge about children’s ‘social environments’ and barriers to collecting data from this group (NCCARF, 2011), as part of an emphasis on using data collection mechanisms to develop the evidence base and meet specific performance indicators for health sector adaptation (DH, 2010; NCCARF, 2011). However, the dominant discourse works to normalise the view that the data are necessarily emergent as are even conceptualisations of the dynamics involved (FGG, 2008).
Socioeconomically disadvantaged groups	380	Germany (17); Wales (12); UK health (10); UK (6); Finland (6); Wales health (6); Scotland health (3); Scotland health (3); Australia health (2); Spain (2); Denmark (2); Belgium (2); Russia (1); Australia (1); Scotland (1)(total =71)	The dominant discourse about socioeconomically disadvantaged groups is a general language suggesting that climate change will increase poverty through its socioeconomic impacts [35], particularly in developing countries [40, 37, 53]. This discourse ostensibly argues mitigation must not increase poverty [51, 52]. It is a discourse not informed by a well-developed framework of understanding of the socioeconomic dimensions of climate change and health. Where poverty is mentioned in relation to specific climate-vulnerable groups, this is in lists of example impacts, with children more often mentioned as the most socioeconomically vulnerable group [53].
Aboriginal people	395	Australia health (35); UK health (11); Australia (5); Germany (3); Belgium (2); Scotland health (1); Scotland (1); Wales (1); UK (1); Finland (1)(total =64)	The dominant discourse is defined by a single country (Australia) with a focus on impacts on Aboriginal people and exhortations to consult with them and other vulnerable groups in developing research [35, 36]. This discourse works to normalise the absence of nuanced policy strategies by representing the adaptive knowledge and resilience of these groups as an unknown, albeit a priority, research question [36]. The USA document is entirely silent about Indigenous vulnerability although it refers to other groups [46].
Rural communities	1186	UK (66); Australia health (39); Germany (19); Finland (12); UK health (10); Netherlands (10); Wales (9); Australia (5); Spain (2); Scotland (3); Wales health (4); Belgium (3); Scotland health (1) (total =183)	The dominant discourse is one of sustainable rural development for both mitigation and adaptation through management of natural assets (woodlands and water) as well as development of agricultural land use and transport infrastructure [34, 39–41, 47, 48, 51–53]. Even in the one Australian health document distinguished by its emphasis on mental health, as well as social cohesion and resilience in rural communities, this discourse is not articulated within a framework of *rural health* vulnerability [36], presenting limited information on rural adaptive assets (COAG, 2007).
Mental health	5334	USA health (11); Australia health (309); Scotland health (66); Wales health (136); UK health (180); Australia (17); Spain (10); Belgium (17); Germany (31); Denmark (14); Wales (25); Finland (66); Scotland (8);UK (45); Russia (3); Netherlands (2)(total =940)	The dominant discourse about mental health most often refers to the wider set of vulnerable groups, reflecting the use of lists to refer to climate vulnerable groups in these documents. Although mental health is elaborated in most detail in the language of rural mental health effects [36], it is almost never translated into nuanced strategies for adaptation [36, 53]. This discourse tends not to be informed by any detail on different categories of mental health conditions beyond depression from extreme weather events effects such as cold, dislocation from flooding, and climate anxiety or a kind of generalised fear for the future [36, 49]. References to the mental health of Aboriginal people do not go beyond linking their holistic relationship to their land to their mental health climate vulnerability and lack detail on consultation strategies with mental health stakeholders generally [36]. With few exceptions [49], this discourse does not move beyond populist concepts of mental health climate vulnerability with little specific guidance on mental health interventions [36].

## Conclusions

This study was predicated on the assumption that an analysis of best practice in health adaptation policy documents involves consideration of the extent to which they offer guidance on the special needs of particular vulnerable groups. The alternative assumption—that policy need not refer to specific climate vulnerable groups on the basis that their needs can be equally well met by broad policy frameworks—is much more difficult to sustain. For example, our previous analyses of accounts of flood management offered some evidence that the groups we have studied here have distinctive needs important to the effective management of climate disasters: early warning systems need to take special account of the needs of culturally and linguistically diverse communities; planning for evacuation centres does need to account for socioeconomically disadvantaged children and people with disabilities, and so on [18]. Of course, there are policy documents such as ‘Clean Air’ regulations that will benefit diverse groups—this study does not at all negate that phenomenon—though the extent to which such documents optimally meet special needs may be unproven. The existence of ostensibly successful policy documents that are silent on the special needs of particular groups is not an argument against developing ‘best practice’ health adaptation policy that explicitly prioritises the needs of vulnerable groups, if the evidence of the IPCC about climate vulnerability and social inequality is to be heeded [11].

This study suggests that health adaptation policy documents do not offer a well-developed basis for implementing national policy for the climate vulnerable groups in this study. The primary Leximancer analysis found that references to climate vulnerable groups are relatively little present, as well as poorly connected to language about practical strategies and socio-economic contexts, both also little present. These disconnects were found even in the smaller body of health references emphasising these vulnerable groups. The qualitative descriptions of the dominant discourses in references to each vulnerable group extended this finding by suggesting that these references involve ‘lists’ of vulnerable groups and crude populist constructions rather than informed policy guidance. This is broadly consistent with other international evidence. The UN national communications and the OECD’s work generally, as well as recent scholarly reviews of the field [6], suggest that while developed nations have made some progress in understanding the impacts of climate projections and identification of adaptation options, much less progress has been made in establishing mechanisms for implementing such adaptation options, including through policy instruments. Negligible adaptation is occurring in practice for climate vulnerable groups. There is known poor reporting and visibility of adaptation activities, notably in the health sector [6]. In health, the challenges are known to lie in the absence of both theoretical (development of aims) and operational (mechanisms for implementation) aspects of adaptation [3, 6, 70]. This study contributes to this body of work by suggesting exactly where and how the needs of climate vulnerable groups are not being met.

How should policy be developed for climate vulnerable groups? The discussion of best practice that follows aims to explore the implications of our findings in ways that have practical value for policy-makers. Given that our study has diagnosed policy inadequacies, discussion of its implications must therefore involve a discussion of what a best practice health adaptation policy document might look like. This discussion is considered by us to be an ethical requirement of our study: we are uncomfortable ethically with diagnosing the inadequacies of policies without also offering some practical suggestions, based on the emerging literature, about best practice policy.

**Figure 2:**
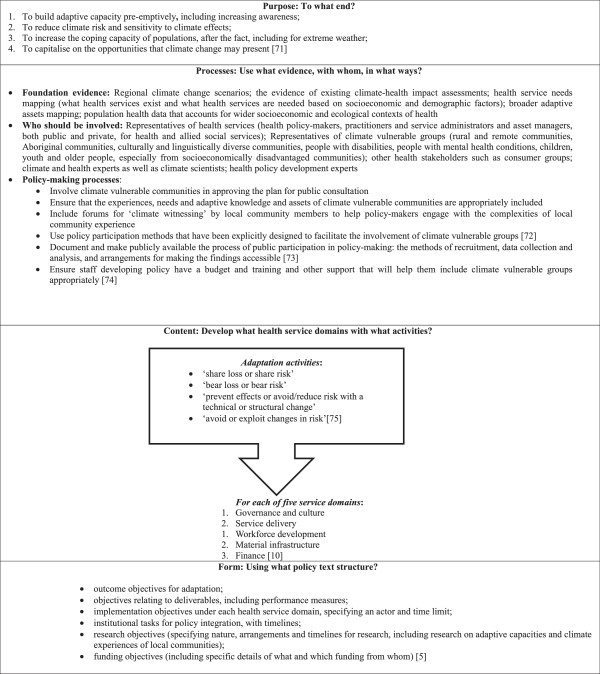
**Available best practice elements for developing national adaptation policy, including for climate vulnerable groups, as part of a social determinants of health approach,**[5, 9, 70–74].

A diverse new body of applied and scholarly literature offers some guidance on best practice in developing adaptation policy, particularly for climate vulnerable groups. Figure 2 summarises this literature, providing the available elements of a best practice approach for developing national adaptation policy informed by a focus on vulnerable groups—in ways that are consistent with the definition of climate vulnerability and the prescriptions of global policy frameworks discussed in the background section of this paper. It offers national adaptation policy objectives; types of foundational evidence for national adaptation policy; policy processes for involving climate vulnerable groups, including through participative methods such as ‘climate witnessing’; exemplars of the content of this policy (health service domains for organising this policy) and the repertoires of adaptive activities that could be delivered under such domains; the form or structure of national adaptation policy into which the health service domains can be integrated in the detail of implementation objectives. Yet it must be acknowledged that this is an emerging field and while the table contains many ‘commonsense approaches’ the evidence for ‘what works’ has yet to be produced in many areas of health adaptation practice,

In an age in which leaders of thinking on public health policy have argued that narrow conceptualisations of policy serve narrow economic interests at odds with a social determinants of health approach [75], health adaptation policy still presents an opportunity. The opportunity is to ensure that the policies of the future and revisions of the immediate past more authentically serve the needs of climate vulnerable communities—in ways that are informed by rich, contextualised strategies and evidence-based theory on the primary social determinants of health.
